# Supplementary material for: Parous rate and longevity of anophelines mosquitoes in bure district, northwestern Ethiopia
Source: PLoS One. 2022 Feb 4;17(2):e0263295. doi: 10.1371/journal.pone.0263295 (PMC8815865; doi:10.1371/journal.pone.0263295)
Supplement: S1 File — (DOCX) [file pone.0263295.s001.docx]

1. **Row data for figure 1**

| Overall percentage of parous rate of Anopheles species across month | | | | | | |
| --- | --- | --- | --- | --- | --- | --- |
| Month | *An. arabiensis* | *An.*  *pharoenis* | *An.*  *funestus* | *An.*  *coustani* | *An.*  *squamosus* | *An. cinereus* |
| July | 30.8 | 100 | 33.3 | 0 | 0 | 0 |
| Aug | 46.7 | 66.6 | 33.3 | 27.3 | 25 | 0 |
| Sept | 44.4 | 0 | 72 | 53.8 | 68.4 | 0 |
| Oct | 62.5 | 0 | 72.4 | 93.6 | 72.7 | 0 |
| Nov | 43.4 | 0 | 30 | 32.4 | 12.5 | 0 |
| Dec | 54.8 | 0 | 22.2 | 20.5 | 28.6 | 0 |
| Jan | 55.3 | 0 | 36.4 | 15 | 20 | 0 |
| Feb | 56.1 | 0 | 42.9 | 20 | 0 | 0 |
| Mar | 68.9 | 0 | 83.9 | 25 | 40 | 27.3 |
| Apr | 47.5 | 0 | 71.4 | 0 | 0 | 33.3 |
| May | 35.5 | 0 | 33.3 | 0 | 28.6 | 50 |
| June | 0 | 0 | 33.3 | 0 | 25 | 25 |

1. **Row data for figure 2 (totally, they are six tables)**

| Proportion of Parous Rate per village (*An. arabiensis*) | | | |
| --- | --- | --- | --- |
|  | Bukta  (Irrigated village) | Workmidr (Non-Irrigated village) | Shnebekuma (Non-irrigated village) |
| July | 0 | 50 | 33.3 |
| Aug | 0 | 50 | 44.4 |
| Sept | 25 | 42.9 | 50 |
| Oct | 20 | 44.4 | 76.9 |
| Nov | 0 | 20 | 50 |
| Dec | 20 | 40 | 75 |
| Jan | 0 | 42.9 | 60 |
| Feb | 33.3 | 0 | 59.5 |
| Mar | 42.2 | 0 | 80.6 |
| April | 50 | 0 | 53.6 |
| May | 40 | 50 | 46.4 |
| June | 0 | 0 | 0 |

| Proportion of parous rate per village  (*An. phareonsis*) | | | |
| --- | --- | --- | --- |
| Month | Bukta | Workmidr | Shnebekuma |
| July | 100 | 0 | 100 |
| Aug | 100 | 0 | 50 |
| Sept | 0 | 0 | 0 |
| Oct | 0 | 0 | 0 |
| Nov | 0 | 0 | 0 |
| Dec | 0 | 0 | 0 |
| Jan | 0 | 0 | 0 |
| Feb | 0 | 0 | 0 |
| Mar | 0 | 0 | 0 |
| April | 0 | 0 | 0 |
| May | 0 | 0 | 0 |
| June | 0 | 0 | 0 |

| Proportion of Parous Rate per villages (*An. funestus*) | | | |
| --- | --- | --- | --- |
| Month | Bukta | Workmidr | Shnebekuma |
| July | 0 | 0 | 50 |
| Aug | 0 | 50 | 20 |
| Sept | 33.3 | 25 | 88.9 |
| Oct | 33.3 | 0 | 76.1 |
| Nov | 0 | 0 | 30 |
| Dec | 0 | 0 | 22.2 |
| Jan | 33.3 | 33.3 | 40 |
| Feb | 0 | 0 | 42.9 |
| Mar | 33.3 | 0 | 89.3 |
| April | 33.3 | 0 | 77.8 |
| May | 0 | 0 | 33.3 |
| June | 0 | 0 | 33.3 |

| Proportion of parous rate per village (*An. coustani*) | | | |
| --- | --- | --- | --- |
| Month | Bukta | Workmidr | Shnebekuma |
| July | 0 | 0 | 0 |
| Aug | 0 | 27.3 | 0 |
| Sept | 33.3 | 37.5 | 72 |
| Oct | 80 | 95.5 | 95 |
| Nov | 20 | 20 | 40.9 |
| Dec | 20 | 20 | 20.7 |
| Jan | 0 | 0 | 15 |
| Feb | 12.5 | 0 | 23.5 |
| Mar | 33.3 | 0 | 16.7 |
| April | 0 | 0 | 0 |
| May | 0 | 0 | 0 |
| June | 0 | 0 | 0 |

| Proportion of parous rate per villages (*An. squamosus*) | | | |
| --- | --- | --- | --- |
| Month | Bukta | Workmidr | Shnebekuma |
| July | 0 | 0 | 0 |
| Aug | 0 | 0 | 25 |
| Sept | 50 | 0 | 70.5 |
| Oct | 50 | 50 | 77.8 |
| Nov | 0 | 0 | 12.5 |
| Dec | 0 | 0 | 28.6 |
| Jan | 0 | 0 | 20 |
| Feb | 0 | 0 | 0 |
| Mar | 0 | 0 | 40 |
| April | 0 | 0 | 0 |
| May | 0 | 28.6 | 0 |
| June | 0 | 25 | 0 |

| Proportion of parous rate per village (*An. cinereus*) | | | |
| --- | --- | --- | --- |
| Month | Bukta | Workmidr | Shnebekuma |
| July | 0 | 0 | 0 |
| Aug | 0 | 0 | 0 |
| Sept | 0 | 0 | 0 |
| Oct | 0 | 0 | 0 |
| Nov | 0 | 0 | 0 |
| Dec | 0 | 0 | 0 |
| Jan | 0 | 0 | 0 |
| Feb | 0 | 0 | 0 |
| Mar | 27.3 | 0 | 0 |
| Apr | 33.3 | 0 | 0 |
| May | 50 | 0 | 0 |
| June | 25 | 0 | 0 |

1. **Row data for table 3 (three), i.e., distribution of six species in three villages**

| Proportion of Parous Rate per village (*An. arabiensis*) | | | |
| --- | --- | --- | --- |
|  | Bukta | Workmidr | Shnebekuma |
| July | 0 | 50 | 33.3 |
| Aug | 0 | 50 | 44.4 |
| Sept | 25 | 42.9 | 50 |
| Oct | 20 | 44.4 | 76.9 |
| Nov | 0 | 20 | 50 |
| Dec | 20 | 40 | 75 |
| Jan | 0 | 42.9 | 60 |
| Feb | 33.3 | 0 | 59.5 |
| Mar | 42.2 | 0 | 80.6 |
| April | 50 | 0 | 53.6 |
| May | 40 | 50 | 46.4 |
| June | 0 | 0 | 0 |

| Proportion of parous rate per village  (*An. phareonsis*) | | | |
| --- | --- | --- | --- |
| Month | Bukta | Workmidr | Shnebekuma |
| July | 100 | 0 | 100 |
| Aug | 100 | 0 | 50 |
| Sept | 0 | 0 | 0 |
| Oct | 0 | 0 | 0 |
| Nov | 0 | 0 | 0 |
| Dec | 0 | 0 | 0 |
| Jan | 0 | 0 | 0 |
| Feb | 0 | 0 | 0 |
| Mar | 0 | 0 | 0 |
| April | 0 | 0 | 0 |
| May | 0 | 0 | 0 |
| June | 0 | 0 | 0 |

| Proportion of Parous Rate per villages (*An. funestus*) | | | |
| --- | --- | --- | --- |
| Month | Bukta | Workmidr | Shnebekuma |
| July | 0 | 0 | 50 |
| Aug | 0 | 50 | 20 |
| Sept | 33.3 | 25 | 88.9 |
| Oct | 33.3 | 0 | 76.1 |
| Nov | 0 | 0 | 30 |
| Dec | 0 | 0 | 22.2 |
| Jan | 33.3 | 33.3 | 40 |
| Feb | 0 | 0 | 42.9 |
| Mar | 33.3 | 0 | 89.3 |
| April | 33.3 | 0 | 77.8 |
| May | 0 | 0 | 33.3 |
| June | 0 | 0 | 33.3 |

| Proportion of parous rate per village (*An. coustani*) | | | |
| --- | --- | --- | --- |
| Month | Bukta | Workmidr | Shnebekuma |
| July | 0 | 0 | 0 |
| Aug | 0 | 27.3 | 0 |
| Sept | 33.3 | 37.5 | 72 |
| Oct | 80 | 95.5 | 95 |
| Nov | 20 | 20 | 40.9 |
| Dec | 20 | 20 | 20.7 |
| Jan | 0 | 0 | 15 |
| Feb | 12.5 | 0 | 23.5 |
| Mar | 33.3 | 0 | 16.7 |
| April | 0 | 0 | 0 |
| May | 0 | 0 | 0 |
| June | 0 | 0 | 0 |

| Proportion of parous rate per villages (*An. squamosus*) | | | |
| --- | --- | --- | --- |
| Month | Bukta | Workmidr | Shnebekuma |
| July | 0 | 0 | 0 |
| Aug | 0 | 0 | 25 |
| Sept | 50 | 0 | 70.5 |
| Oct | 50 | 50 | 77.8 |
| Nov | 0 | 0 | 12.5 |
| Dec | 0 | 0 | 28.6 |
| Jan | 0 | 0 | 20 |
| Feb | 0 | 0 | 0 |
| Mar | 0 | 0 | 40 |
| April | 0 | 0 | 0 |
| May | 0 | 28.6 | 0 |
| June | 0 | 25 | 0 |

| Proportion of parous rate per village (*An. cinereus*) | | | |
| --- | --- | --- | --- |
| Month | Bukta | Workmidr | Shnebekuma |
| July | 0 | 0 | 0 |
| Aug | 0 | 0 | 0 |
| Sept | 0 | 0 | 0 |
| Oct | 0 | 0 | 0 |
| Nov | 0 | 0 | 0 |
| Dec | 0 | 0 | 0 |
| Jan | 0 | 0 | 0 |
| Feb | 0 | 0 | 0 |
| Mar | 27.3 | 0 | 0 |
| Apr | 33.3 | 0 | 0 |
| May | 50 | 0 | 0 |
| June | 25 | 0 | 0 |
