# Supplementary material for: Parous rate and longevity of anophelines mosquitoes in bure district, northwestern Ethiopia
Source: PLoS One. 2022 Feb 4;17(2):e0263295. doi: 10.1371/journal.pone.0263295 (PMC8815865; doi:10.1371/journal.pone.0263295)
Supplement: Attachment — Submitted filename: Response to Reviewers.docx [file pone.0263295.s002.docx]

**Response letter**

**Comment 1: Prepare based on PLOS ONE journal guideline**

**Response** 1: We have tried to prepare the manuscript based on the PLOS ONE journal guideline

criteria.

**Comment 2: Regarding “ethical statement”**

**Response 2**: As indicated by the reviewer / editor, ethical statements are included under materials

and methods section. In particular, next to data analysis. It is highlighted in the

manuscript.

**Comment 3: About “Funding Information’ and ‘Financial Disclosure”**

**Response 3**: We didn’t get any fund source from any organization. Being this, we clearly indicated

in the that “The author(s) received no specific funding for this work” in the funding

information section.

**Comment 4. About data access / provision.**

**Response 4:** I corrected myself. It is possible to access any necessary data /no restriction/ if you

require. Being this, revision of the cover letter is not necessary because there are

no data restrictions. All necessary data for this manuscript is already uploaded. If

the reviewer is required raw data; I can do now.

**Comment 5: About ethical statement/ repeated/**

**Response 5**: Enough information about ethical statements is treated under material method

section. All available and required information are incorporated.

**Comment 6: Regarding references lists, i.e., review your reference list to ensure that it is**

**complete and correct.**

**Response 6:** All used references are cited in the manuscript and follow the correct ways of

referencing style. There is no new citation and reference add in the manuscript.

- **Additional Editor Comments**

**Comment**: Please also review the sentence "Usually, older females also have higher

exposure rates..................It is indicated in the introduction.

**Response:** If you see the truth, naturally female known by feeding blood meals from

various animals including human to mature the fertilized eggs. Until she gets

die, she will have many batches of eggs. For each batches, she will take 2-3

blood meals. Therefore, to have many batches of eggs, the female should take

many times of blood meals. This increase the rate of exposure to be infected by

the parasites. Because of having this kinds of truth, we prefer to use the phrase

“usually, older females also have higher exposure rates….”.

In conclusion, the phrase hasn’t any problem /it is science/

- **Comment:** About calculating the life expectancy of mosquitoes

Response: Partly, I agree with your saying, but we have no any other option to do it. In

similar fashion (objective), the following researchers have used the formal

driven by Davidson (1954) to calculate mosquito life expectancy (longevity).

**[34]** Ndoen, E., Wild, C., Dale, P., Sipe, N., Dale, M. (2012). Mosquito Longevity, Vector

Capacity, and Malaria Incidence in West Timor and Central Java, Indonesia.

International Scholarly Research Network. doi:10.5402/2012/143863.

[**62**] Taye B, Lelisa K, Emana D, Asale A, Yewhalaw D. Seasonal dynamics, longevity and

biting activity of anopheline mosquitoes in southwest Ethiopia. J Insect Sci.

2016;16:1-7. doi: 10.1093/jisesa/iev150

Therefore, we can say that we are correct and haven’t any choice.

**Comments: To prepare figures using PACE**

**Response**: I did it after I registered. The uploaded figures are developed using the given

comments. It meets the requirement of PLOS ONE criteria, based on PACE.
